# Genome-Wide Association Study of Retinopathy in Individuals without Diabetes

**DOI:** 10.1371/journal.pone.0054232

**Published:** 2013-02-05

**Authors:** Richard A. Jensen, Xueling Sim, Xiaohui Li, Mary Frances Cotch, M. Kamran Ikram, Elizabeth G. Holliday, Gudny Eiriksdottir, Tamara B. Harris, Fridbert Jonasson, Barbara E. K. Klein, Lenore J. Launer, Albert Vernon Smith, Eric Boerwinkle, Ning Cheung, Alex W. Hewitt, Gerald Liew, Paul Mitchell, Jie Jin Wang, John Attia, Rodney Scott, Nicole L. Glazer, Thomas Lumley, Barbara McKnight, Bruce M. Psaty, Kent Taylor, Albert Hofman, Paulus T. V. M. de Jong, Fernando Rivadeneira, Andre G. Uitterlinden, Wan-Ting Tay, Yik Ying Teo, Mark Seielstad, Jianjun Liu, Ching-Yu Cheng, Seang-Mei Saw, Tin Aung, Santhi K. Ganesh, Christopher J. O'Donnell, Mike A. Nalls, Kerri L. Wiggins, Jane Z. Kuo, The Blue Mountains Eye Study GWAS team, CKDGen Consortium, Cornelia M. van Duijn, Vilmundur Gudnason, Ronald Klein, David S. Siscovick, Jerome I. Rotter, E. Shong Tai, Johannes Vingerling, Tien Y. Wong

**Affiliations:** 1 Cardiovascular Health Research Unit, University of Washington, Seattle, Washington, United States of America; 2 Department of Medicine, University of Washington, Seattle, Washington, United States of America; 3 Centre for Molecular Epidemiology, National University of Singapore, Singapore, Singapore; 4 Medical Genetics Institute, Cedars-Sinai Medical Center, Los Angeles, California, United States of America; 5 Division of Epidemiology and Clinical Applications, National Eye Institute, Intramural Research Program, National Institutes of Health, Bethesda, Maryland, United States of America; 6 Singapore Eye Research Institute, Singapore National Eye Centre, Singapore, Singapore; 7 Department of Ophthalmology, Erasmus Medical Center, Rotterdam, The Netherlands; 8 School of Medicine and Public Health, University of Newcastle, Newcastle, New South Wales, Australia; 9 Icelandic Heart Association, Kopavogur, Iceland; 10 Laboratory of Epidemiology, Demography, and Biometry, National Institute on Aging, Intramural Research Program, National Institutes of Health, Bethesda, Maryland, United States of America; 11 Department of Medicine, University of Iceland, Reykjavik, Iceland; 12 Department of Ophthalmology, Landspitalinn University Hospital, Reykjavik, Iceland; 13 Department of Ophthalmology and Visual Science, School of Medicine and Public Health, University of Wisconsin, Madison, Wisconsin, United States of America; 14 Human Genetics Center and Institute of Molecular Medicine, University of Texas Health Science Center at Houston, Houston, Texas, United States of America; 15 Centre for Eye Research Australia, University of Melbourne, East Melbourne, Victoria, Australia; 16 Centre for Vision Research, Department of Ophthalmology and the Westmead Millennium Institute, University of Sydney, Sydney, Australia; 17 Department of Medicine, John Hunter Hospital and Hunter Medical Research Institute, Newcastle, New South Wales, Australia; 18 School of Biomedical Sciences, University of Newcastle, Newcastle, New South Wales, Australia; 19 Section of Preventive Medicine and Epidemiology, Boston University School of Medicine, Boston, Massachusetts, United States of America; 20 Department of Statistics, University of Auckland, Auckland, New Zealand; 21 Department of Biostatistics, University of Washington, Seattle, Washington, United States of America; 22 Department of Epidemiology, University of Washington, Seattle, Washington, United States of America; 23 Department of Health Services, University of Washington, Seattle, Washington, United States of America; 24 Group Health Research Institute, Group Health Cooperative, Seattle, Washington, United States of America; 25 Department of Epidemiology, Erasmus Medical Center, Rotterdam, The Netherlands; 26 Netherlands Institute of Neuroscience, Amsterdam, The Netherlands; 27 Department of Ophthalmology, Academic Medical Center, Amsterdam, The Netherlands; 28 Department of Internal Medicine, Erasmus Medical Center, Rotterdam, The Netherlands; 29 Department of Clinical Chemistry, Erasmus Medical Center, Rotterdam, The Netherlands; 30 Department of Epidemiology and Public Health, National University of Singapore, Singapore, Singapore; 31 Department of Statistics and Applied Probability, National University of Singapore, Singapore, Singapore; 32 NUS Graduate School for Integrative Science and Engineering, National University of Singapore, Singapore, Singapore; 33 Genome Institute of Singapore, Agency for Science, Technology and Research, Singapore, Singapore; 34 Institute of Human Genetics, University of California San Francisco, San Francisco, California United States of America; 35 Department of Ophthalmology, National University of Singapore, Singapore, Singapore; 36 Division of Cardiovascular Medicine, Department of Internal Medicine, University of Michigan, Ann Arbor, Michigan, United States of America; 37 National Heart, Lung, and Blood Institute’s Framingham Heart Study, Framingham, Massachusetts, United States of America; 38 Division of Intramural Research, National Heart, Lung, and Blood Institute, Bethesda, Maryland, United States of America; 39 Cardiology Division, Massachusetts General Hospital, Harvard Medical School, Boston, Massachusetts, United States of America; 40 Laboratory of Neurogenetics, Intramural Research Program, National Institute on Aging, National Institutes of Health, Bethesda, Maryland, United States of America; 41 Department of Medicine, National University of Singapore, Singapore, Singapore; 42 Duke-National University of Singapore Graduate Medical School, Singapore, Singapore; Sanjay Gandhi Medical Institute, India

## Abstract

**Background:**

Mild retinopathy (microaneurysms or dot-blot hemorrhages) is observed in persons without diabetes or hypertension and may reflect microvascular disease in other organs. We conducted a genome-wide association study (GWAS) of mild retinopathy in persons without diabetes.

**Methods:**

A working group agreed on phenotype harmonization, covariate selection and analytic plans for within-cohort GWAS. An inverse-variance weighted fixed effects meta-analysis was performed with GWAS results from six cohorts of 19,411 Caucasians. The primary analysis included individuals without diabetes and secondary analyses were stratified by hypertension status. We also singled out the results from single nucleotide polymorphisms (SNPs) previously shown to be associated with diabetes and hypertension, the two most common causes of retinopathy.

**Results:**

No SNPs reached genome-wide significance in the primary analysis or the secondary analysis of participants with hypertension. SNP, rs12155400, in the histone deacetylase 9 gene (*HDAC9*) on chromosome 7, was associated with retinopathy in analysis of participants without hypertension, −1.3±0.23 (beta ± standard error), p = 6.6×10^−9^. Evidence suggests this was a false positive finding. The minor allele frequency was low (∼2%), the quality of the imputation was moderate (r^2^ ∼0.7), and no other common variants in the *HDAC9* gene were associated with the outcome. SNPs found to be associated with diabetes and hypertension in other GWAS were not associated with retinopathy in persons without diabetes or in subgroups with or without hypertension.

**Conclusions:**

This GWAS of retinopathy in individuals without diabetes showed little evidence of genetic associations. Further studies are needed to identify genes associated with these signs in order to help unravel novel pathways and determinants of microvascular diseases.

## Introduction

Mild retinopathy, defined as the presence of isolated microaneurysms or dot-blot hemorrhages, is frequently observed in the general non-diabetic population. Several population studies report these signs are found in 5 to 15% of adults without diabetes [1], [2], [3]. These retinopathy lesions represent a disruption of the blood–retinal barrier that results from increased arteriolar tone, vasospasm, media hyperplasia, intimal thickening and degeneration of vascular smooth muscle with or without endothelial cell necrosis [3]. In addition, these clinical findings are typically transitory in nature making them difficult to study [2].

The etiology of mild retinopathy in individuals without diabetes remains unclear [4]. Risk factors identified in individuals without diabetes include older age, hypertension, impaired glucose tolerance, dyslipidemia, obesity and elevated levels of inflammatory markers. Of these, hypertension is the major risk factor [5], [6], [7], [8], [9]. In normotensive individuals, few risk factors have been consistently identified.

Importantly, mild retinopathy signs have been suggested to reflect microvascular disease in other end organs. For example, studies have shown mild retinopathy signs are associated with renal dysfunction [10], [11], [12], incident hypertension [1], clinical stroke [13], [14], [15], [16], [17], congestive heart failure [18], and cardiovascular mortality [19], [20]. Mild retinopathy may also signal subsequent risk of clinical diabetes for individuals who do not currently have diabetes but have a family history of diabetes [21].

To date, no genetic studies of mild retinopathy have been conducted in a population without diabetes. In an attempt to identify biological pathways involved in the pathogenesis of these signs, we conducted a genome-wide association study (GWAS) to examine the association between ∼2.5 million single nucleotide polymorphisms (SNPs) and the presence of isolated microaneurysms or dot-blot hemorrhages. This was done in six community-based cohorts comprising 19,411 individuals of European ancestry, followed by a meta-analysis of GWAS results. The primary analysis was restricted to individuals without diabetes and secondary analyses were stratified by hypertension status. No European cohorts were available for replication so we examined the SNP and locus transferability of genome-wide significant results in a cohort of Singapore Indian Asians and an African American cohort. We also investigated the association of these loci with other diseases that may share a similar microvascular etiology, including kidney disease, in cohorts of European ancestry. Finally, we looked at SNPs identified in previous GWAS of diabetes and hypertension (the two most common causes of retinopathy [22], [23]) to determine if they were associated with retinopathy in a normotensive population thought to be free of diabetes.

## Methods

### Ethics Statement

Each cohort secured approval from their respective institutional review boards, and all participants provided written informed consent in accordance with the Declaration of Helsinki.

### Study Population

The study population was comprised of individuals from six studies across Australia, Europe and the United States. Participants of European ancestry came from the Cohorts for Heart and Aging Research in Genetic Epidemiology (CHARGE) Consortium [24] {the Age, Gene/Environment Susceptibility (AGES)–Reykjavik Study [25], the Atherosclerosis Risk in Communities (ARIC) Study [26], the Cardiovascular Health Study (CHS) [27], and the Rotterdam Study (RS) [28]}, the Blue Mountain Eye Study [29] (BMES) and the Multi-Ethnic Study of Atherosclerosis (MESA) [30].

All participating studies approved guidelines for collaboration, and a working group agreed on phenotype harmonization, covariate selection and analytic plans for within-study analyses and meta-analyses of results. Details of each participating study are provided in the Supplementary Material including sample selection, retinopathy grading, genotyping platform, imputation algorithm, and quality control procedures used by each study.

The Reykjavik Study is a population study with a random sample of 30,794 residents assembled in 1967 to study cardiovascular disease and its risk factors among those born between 1907 and 1935. The AGES is a prospective study of 5,764 surviving members, aged 66 years and older recruited from 2002–2006 [25]. It was designed to examine genetic susceptibility and environmental interactions as risk factors for disease and disability in old age.

The ARIC study is a population-based prospective cohort study of cardiovascular disease and its risk factors [26]. ARIC included 15,792 individuals aged 45 to 64 years at baseline (1987–1989), selected by probability sampling from four US communities.

The BMES is a population-based cohort study of vision and common eye diseases in an urban older population comprising two postcode areas in the Blue Mountains region, west of Sydney, Australia [29]. The original cohort (1992–1994) included 3,654 participants, of those, 2,335 participants were examined at the five-year follow up exam and 1,952 at the ten-year exam.

The CHS is a population-based cohort study of risk factors for cardiovascular disease in adults 65 years of age or older conducted at four field centers [27]. The original cohort of predominantly European ancestry consisted of 5,201 participants recruited in 1989–1990 from random samples of Medicare lists. An additional 687 African-Americans were enrolled in 1992–1993.

MESA is a prospective study of 6,814 adults aged 45–84 years with no history of clinical cardiovascular disease at the baseline exam (July 2000–July 2002) [30]. Each site recruited equal numbers of men and women with site-specific racial and ethnic proportions. Participants defined themselves as European, African, Hispanic, or Chinese Americans. Only subjects of European ancestry were used in the discovery phase of this study.

The RS is a prospective population-based cohort study comprising 7,983 participants aged 55 years or older from Ommoord, a district of Rotterdam, the Netherlands. It was designed to investigate the incidence and progression of diseases in the elderly and the original cohort was identified between 1990 and 1993 [28].

### Retinopathy

Retinopathy was defined as the presence of microaneurysms or dot-blot hemorrhages. Individuals with diabetes were excluded. We also excluded individuals with secondary causes of retinopathy (e.g. branch or central retinal vein occlusions, late age-related macular degeneration) and other retinopathy phenotypes including exudates without microaneurysms or dot-blot hemorrhages and pre-retinal or vitreous hemorrhages.

Fundus photographs were graded using site-specific standardized protocols (Supplemental Material). Not all sites took pictures with multiple fields of view through dilated pupils in both eyes. Some sites took photographs through undilated pupils (ARIC, CHS, MESA), some sites were limited to photographs using 1 field of view (ARIC, CHS, RS) and one site (CHS) did not take photographs of both eyes.

### Diabetes and Hypertension

Diabetes was defined by self-report or fasting blood glucose ≥126 mg/dL [7.0 mmol/L]. In Rotterdam, fasting glucose was not available at baseline so participants were excluded if random glucose was >198 mg/dL [11.0 mmol/L]. Hypertension was defined as current use of blood pressure medication or systolic/diastolic blood pressure ≥140/90 mm Hg.

### Genotyping

Different genotyping platforms were used by the individual studies prior to formation of collaborative phenotype working groups. The AGES and CHS used the Illumina HumanCNV370-Duo. The ARIC and MESA studies used the Affymetrix GeneChip SNP Array 6.0. The BMES used the Illumina Human670Quadv1 custom chip. The RS used the Illumina Infinium HumanHap550-chip v3.0. All of the cohorts imputed to about 2.5 million SNPs using the HapMap Centre d’Etude du Polymorphisme Humain collection samples from Utah (CEU) reference panel [31] after quality control analyses, (Supplementary Material).

### Statistical Analysis

In participants without diabetes, a GWAS of mild retinopathy including microaneurysms and dot-blot hemorrhages was conducted by each of the six cohorts described above. Logistic regression was performed using an additive genetic model with a 1-degree of freedom test of trend relating the presence of retinopathy to genotype dosage (0–2 copies of the coded allele) and adjusting for age, sex and systolic blood pressure. For individuals currently taking medication to control blood pressure, 10 mm of Hg was added to the measured systolic blood pressure based on methods established for previous GWAS of hypertension and blood pressure [32]. This was chosen as a more parsimonious method rather than adjusting separately for both systolic blood pressure and current use of medication to treat hypertension. ARIC and CHS also adjusted for clinic site, BMES adjusted for four multi-dimensional scaling dimensions and MESA adjusted for population structure using 10 principal components. Regression coefficients and their standard errors were determined using the ProbABEL program (http://mga.bionet.nsc.ru/~yurii/ABEL/) [33] in AGES, ARIC, BMES and RS. R software (http://www.r-project.org) was used in CHS and SNPTEST v2 [34], [35] was used in MESA.

An inverse-variance weighted fixed effects meta-analysis of the beta coefficients and standard errors from each site was performed using METAL software (http://www.sph.umich.edu/csg/abecasis/Metal/index.html). The fixed-effects analysis provides a good approximation of the estimates we would get from a combined analysis in which we adjusted for cohort effects. While a random-effects analysis adjusts for heterogeneity between studies, there were too few cohorts available in this study to have power to measure heterogeneity across cohorts. Strand information was available from all the cohorts and all results were synchronized to the forward strand. The level of genome-wide significance was set at p<5×10^−8^, corresponding to a family-wise p-value of 0.05 after Bonferroni correction for one million independent tests. To account for possible residual population structure or other confounding factors, genomic control [36] was applied to each cohort before meta-analysis. This entailed multiplying the standard error of the SNP regression coefficient by the square root of the estimated genomic control inflation factor for that cohort.

In the secondary analyses, participants were stratified by hypertension status, defined above. Adjustments for the stratified analyses were the same as those used for the primary analysis except no adjustment was included for systolic blood pressure in individuals without hypertension and systolic blood pressure was replaced by an indicator variable for treatment of hypertension (yes/no) in the analyses of individuals with hypertension.

We also examined the SNP and locus transferability of our findings to cohorts of other ethnicities including Singapore Asian Indians from the Singapore Indian Eye Study (SINDI) [37] and African Americans in the MESA cohort. First we looked at the SNP transferability to the SINDI and MESA cohorts using a significance level of 0.05. Second we looked at the locus transferability by examining all the SNPs in the gene of interest. For those tests we determined the number of tag SNPs required to cover the gene of interest for each ethnicity using the Genome Variation Server (http://gvs.gs.washington.edu/GVS). In all cases we excluded HapMap 3 and selected the following additional parameters; minor allele frequency of >0.01, r^2^ threshold = 0.5 and no monomorphic sites. Combined samples and combined variations of the Han Chinese of Beijing China (HCT)/Japanese in Tokyo, Japan (JPT) populations were used for the Singapore cohort and the Yoruba in Ibadan, Nigeria (YRI) population for the African American cohort from MESA. The level of significance was set at the Bonferroni-corrected p-value based on the number of tag SNPs for each population at each gene tested, p<4.9×10^−4^ for Singapore Asian Indians and p<1.7×10^−4^ in African Americans.

In addition, we investigated the association of these loci with chronic kidney disease and cardiovascular disease. This was accomplished by performing in silico look-ups in the Chronic Kidney Disease Genetics (CKDGen) consortium [38] (chronic kidney disease, microalbuminuria, urinary albumin/creatinine ratio, and renal function as estimated by serum creatinine [eGFRcrea]), in the Wellcome Trust Case Control Consortium [39] (coronary artery disease), the CHARGE Hematology Working Group (anemia - defined as hemoglobin <12 g/dl in women, <13 g/dl in men) and the Heart and Vascular Health (HVH) Study [40], [41] (stroke and myocardial infarction). Look-ups in CKDGen were performed in all participants and two subgroups, those without diabetes and those without hypertension.

Lastly we examined our findings for the SNPs that have previously been shown to be associated with diabetes or hypertension in other GWAS [42], [43]. We also examined SNPs associated with diabetic retinopathy in a recently published candidate gene study [44]. The level of significance was set at the Bonferroni-corrected p-value for the total number of SNPs (p<4.8×10^−4^).

Quantile-quantile (QQ-) plots of –log_10_ (observed p-value) versus –log_10_ (expected p-value) and the Manhattan plots were generated using R software (http://www.r-project.org). Regional association and linkage disequilibrium plots were generated using the SNP Annotation and Proxy Search v2.2 [45]. Forest plots were generated using Stata Statistical Software: Release 11. College Station, TX: StataCorp LP. These plots incorporated the I^2^ heterogeneity statistic to measure the percentage of variation attributable to differences in effect size between cohorts [46], [47].

## Results

Population characteristics stratified by hypertension status for each participating cohort are provided in Table 1. The distribution of retinopathy and specific lesions (where available) are provided in Table 2. The primary meta-analysis included all individuals without diabetes from the six cohorts. There were a total of 2,675,979 genotyped or imputed SNPs that passed quality control in one or more cohorts.

**Table 1:** Subject characteristics by study site.

Subject Characteristic	AGES	ARIC	BMES	CHS	MESA	RS
Sample Size*	2,451	7,116	2,237	966	2,059	4,582
HTN	1,941	2,614	1,656	494	728	1,433
No HTN	510	4,502	581	472	1,331	3,149
Age (years)†	76.0±5.4	60.1±5.6	66.8±9.1	78.3±4.1	63.9±10.1	67.9±8.3
HTN	76.5±5.4	61.5±5.6	67.9±8.8	78.5±4.2	67.7±9.6	69.9±8.3
No HTN	74.2±4.9	59.3±5.5	63.8±9.0	78.1±3.9	61.7±9.7	67.1±8.1
Body Mass Index (kg/m^2^)†	26.9±4.3	27.5±4.9	27.5±4.7	26.3±4.1	27.5±4.9	26.2±3.6
HTN	27.1±4.4	28.8±5.3	28.0±4.8	26.9±4.4	28.7±4.9	27.2±3.7
No HTN	26.0±4.0	26.8±4.5	26.1±3.9	25.6±3.5	26.8±4.7	25.8±3.5
Systolic Blood Pressure (mm Hg)†	141.7±19.7	121.9±17.9	146.4±21.5	134.5±20.0	120.2±18.9	138.0±21.8
HTN	145.9±19.6	133.7±19.8	154.6±18.4	137.1±20.7	132.7±20.4	155.6±22.5
No HTN	125.6±9.3	115.0±12.2	123.3±9.5	131.8±18.9	112.7±13.1	130.0±16.1
Diastolic Blood Pressure (mm Hg)†	74.2±9.5	70.8±9.9	84.7±10.2	68.5±10.8	68.8±9.6	73.8±11.3
HTN	75.0±9.9	74.4±11.0	87.3±9.5	68.6±10.0	71.7±10.2	80.0±11.9
No HTN	71.3±7.2	68.6±8.49	77.2±8.1	68.4±11.5	67.0±8.6	71.0±9.8
Females (%)	58.9	54.3	56.6	64.7	52.0	58.6
HTN	59.8	52.8	57.7	65.2	50.4	63.1
No HTN	55.7	55.1	53.5	64.2	52.7	56.5
Current Smokers (%)	12.9	17.4	9.8	5.6	17.1	23.3
HTN	12.1	14.8	8.3	4.9	10.7	19.2
No HTN	15.9	18.9	14.1	6.4	20.8	25.1
Fasting Glucose (mg/dl)	99.1±9.0	98.4±10.0	98.5±30.3	91.5±9.4	91.1±9.4	113.4±23.4‡
HTN	99.6±9.1	100.0±10.3	99.9±30.7	92.7±9.5	92.8±9.8	117.0±23.4‡
No HTN	97.3±8.6	97.4±9.7	94.7±28.7	90.2±9.1	90.0±8.9	111.6±23.4‡

* Sample size of cohort used for primary analysis.

† Presented as mean (standard deviation).

‡ Random glucose. HTN: Current use of blood pressure medication or systolic/diastolic blood pressure ≥140/90. AGES: Age, Gene/Environment Susceptibility-Reykjavik Study. ARIC: Atherosclerosis Risk in Communities study. BMES: Blue Mountains Eye Study. CHS: Cardiovascular Health Study. MESA: Multi-Ethnic Study of Atherosclerosis. RS: Rotterdam Study.

**Table 2:** Distribution of retinopathy lesions.

Cohort	n	Any Retinopathy* (%)	Microaneurysms (%)	Dot-blot Hemorrhages (%)
AGES				
All subjects	2451	11.4	6.3	5.9
HTN	1941	12.0	6.4	6.3
No HTN	510	9.4	5.7	4.1
ARIC				
All subjects	7,116	2.3	1.2	1.3
HTN	2,614	2.8	1.5	1.7
No HTN	4,502	2.1	1.0	1.1
BMES				
All subjects	2,237	8.4	NA	NA
HTN	1,656	9.2	NA	NA
No HTN	581	6.4	NA	NA
CHS				
All subjects	966	5.6	2.8	3.8
HTN	494	6.9	3.9	4.5
No HTN	472	4.2	1.7	3.2
MESA				
All subjects	2,059	7.0	4.2	3.0
HTN	728	8.9	5.1	4.3
No HTN	1,331	6.0	3.7	2.3
RS				
All subjects	4,582	6.4	NA	NA
HTN	1,433	8.6	NA	NA
No HTN	3,149	5.3	NA	NA

* defined as the presence of microaneurysms or dot-blot hemorrhages excluding individuals with self-report of diabetes or fasting blood glucose ≥126 mg/dL [7.0 mmol/L]) and individuals with secondary causes of retinopathy (e.g. branch or central retinal vein occlusions, late age-related macular degeneration) and other retinopathy phenotypes including exudates without microaneurysms or dot-blot hemorrhages and pre-retinal or vitreous hemorrhages. RS defined diabetes as a random glucose > = 11.1 mmol/L, diabetic medication or self-reported history. HTN: Current use of blood pressure medication or systolic/diastolic blood pressure ≥140/90. AGES: Age, Gene/Environment Susceptibility-Reykjavik Study. ARIC: Atherosclerosis Risk in Communities study. BMES: Blue Mountains Eye Study. CHS: Cardiovascular Health Study. MESA: Multi-Ethnic Study of Atherosclerosis. RS: Rotterdam Study. NA: Not Available.

No SNPs reached genome wide significance in the primary analysis adjusted for age, sex, systolic blood pressure and site specific population stratification covariates (study site or principal components), Table S1 and Figures S1 and S2.

The secondary analyses were stratified by hypertension status. Adjustments in the group of participants with hypertension included age, sex, treatment for hypertension (yes/no) and site specific population covariates (study site or principal components). In individuals with hypertension, no SNPs reached genome-wide significant association, Table S2 and Figures S3 and S4.

**Figure 1:**
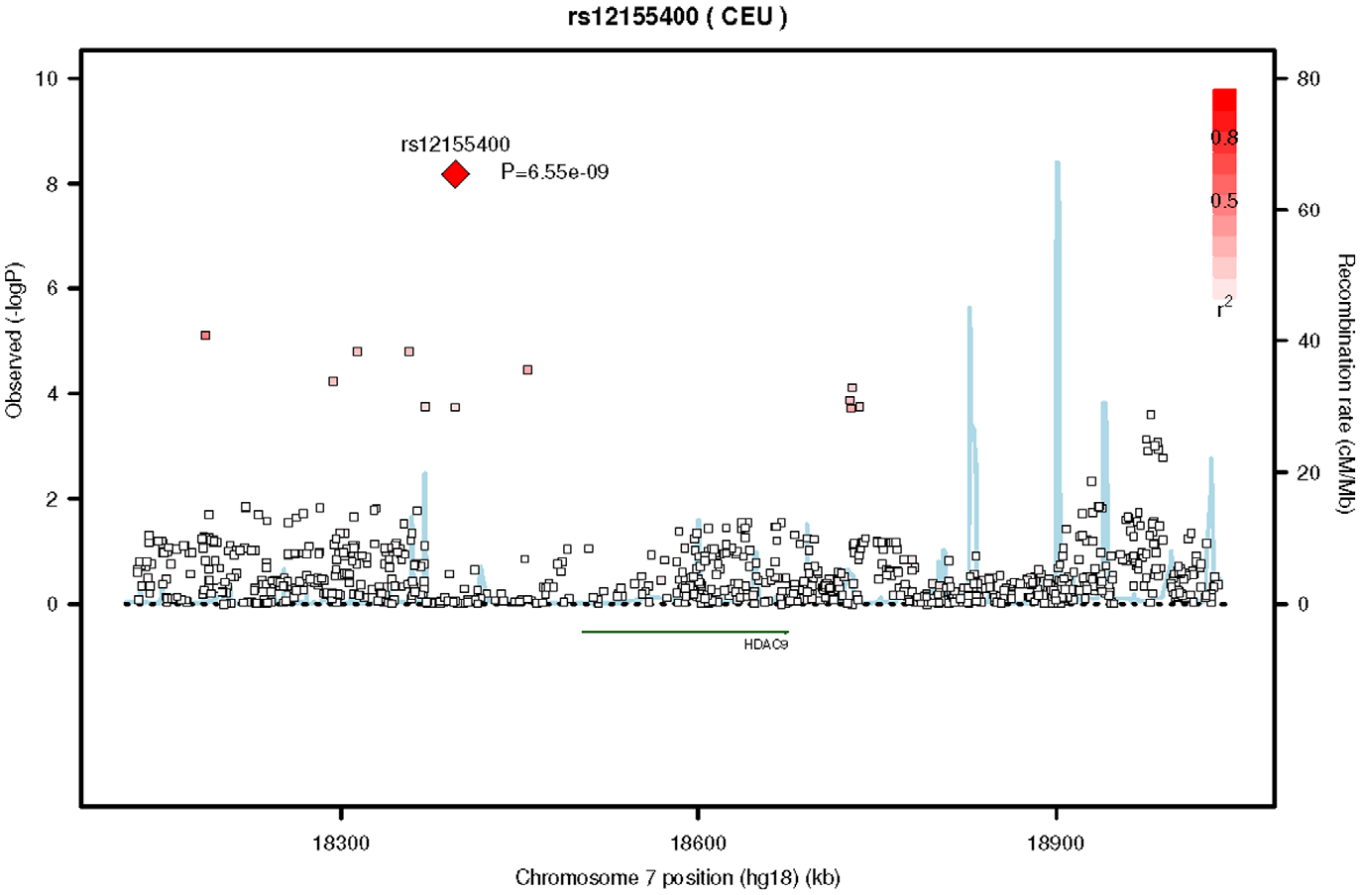
Regional association plot of SNP rs12155400 on chromosome 7 for Caucasians. This figure is the regional association plot of SNP rs12155400 on chromosome 7 that reached genome-wide significance in the meta-analysis of GWAS results in participants of European ancestry without diabetes or hypertension. The lead and surrounding SNPs are color coded according to the pair-wise linkage disequilibrium (LD) with the lead SNP (presented as a diamond) on a scale of r^2^ from 0 to 1. Estimated recombination rates reflect the local LD structure in the 500 kb buffer around the index SNP and plotted based on values from HapMap II Centre d’Etude du Polymorphisme Humain collection samples from a Utah (CEU) population.

**Figure 2:**
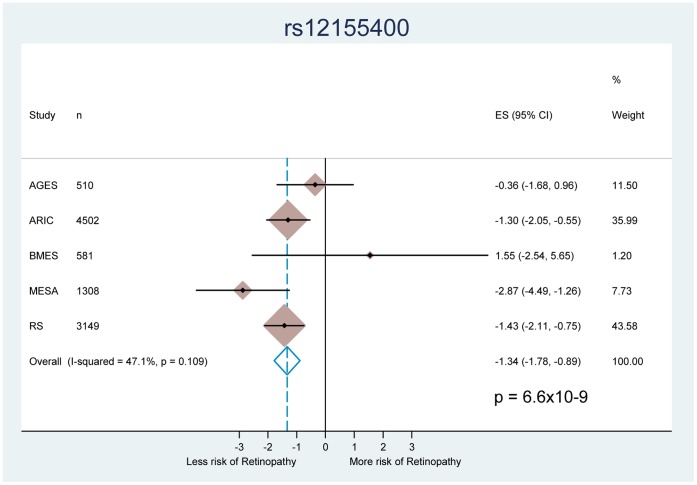
Forest plot. This figure display the direction, effect, 95% confidence interval, sample size and % weight from each individual discovery cohort and overall for the association between SNP rs12155400 on chromosome 7 and retinopathy defined as the presence of microaneurysms or dot-blot hemorrhages. The I^2^ heterogeneity statistic is a measure of the percentage of variation attributable to differences in effect sizes between cohorts. Results from this SNP were not available in CHS.

The analysis of the group of participants without hypertension was adjusted for age, sex and site specific population stratification covariates (study site or principal components), Table S3 and Figures S5 and S6. One SNP, rs12155400, in the histone deacetylase 9 gene (*HDAC9*) on chromosome 7, reached genome-wide significance, −1.3±0.23 (beta ± standard error), p = 6.6×10^−9^, Figure 1 and 2. This was an imputed SNP of relatively low minor allele frequency (MAF) around 2%. The observed divided by the expected variance for imputed allele dosage, a measure of imputation quality, was 0.68, 0.64 and 0.70 for AGES, MESA and BMES respectively. Imputation quality in ARIC and RS was measured by r^2^ and was 0.71 and 0.74, respectively.

There were 849 SNPs in the meta-analysis in proximity to the reference gene *HDAC9*. Eight of those SNPs were in linkage disequilibrium (LD) with SNP rs12155400 (r^2^ ranged from 0.10 to 0.33). Only 1 of those 8 SNPs was genotyped in any cohort, rs10225230, which was genotyped in ARIC and MESA. The r^2^ between rs12155400 and rs10225230 was 0.33 and their positions are separated by 332 K base pairs. The results for the imputed SNP rs12155400 and the genotyped SNP rs10225230 were exactly the same in the two cohorts that genotyped rs10225230 with a p-value of 0.03 and 0.003 for MESA and ARIC respectively, indicating the results were not due to poor imputation quality at rs12155400 in these two cohorts. The overall p-value at rs10225230 was 1.9×10^−4^. The minor allele frequencies were similar, 0.02 for rs12155400 and 0.03 for rs10225230.

The SNP with the highest imputation quality that was in LD with rs12155400 was rs10234685. The imputation quality ranged from 0.88 to 0.99. The r^2^ between rs12155400 and rs10234685 was 0.10 and those 2 SNPs were separated by 386,000 base pairs. Mild retinopathy was not associated with SNP rs10234685, p-value = 0.20. The minor allele frequency for rs10234685 was 0.07.

**Figure 3:**
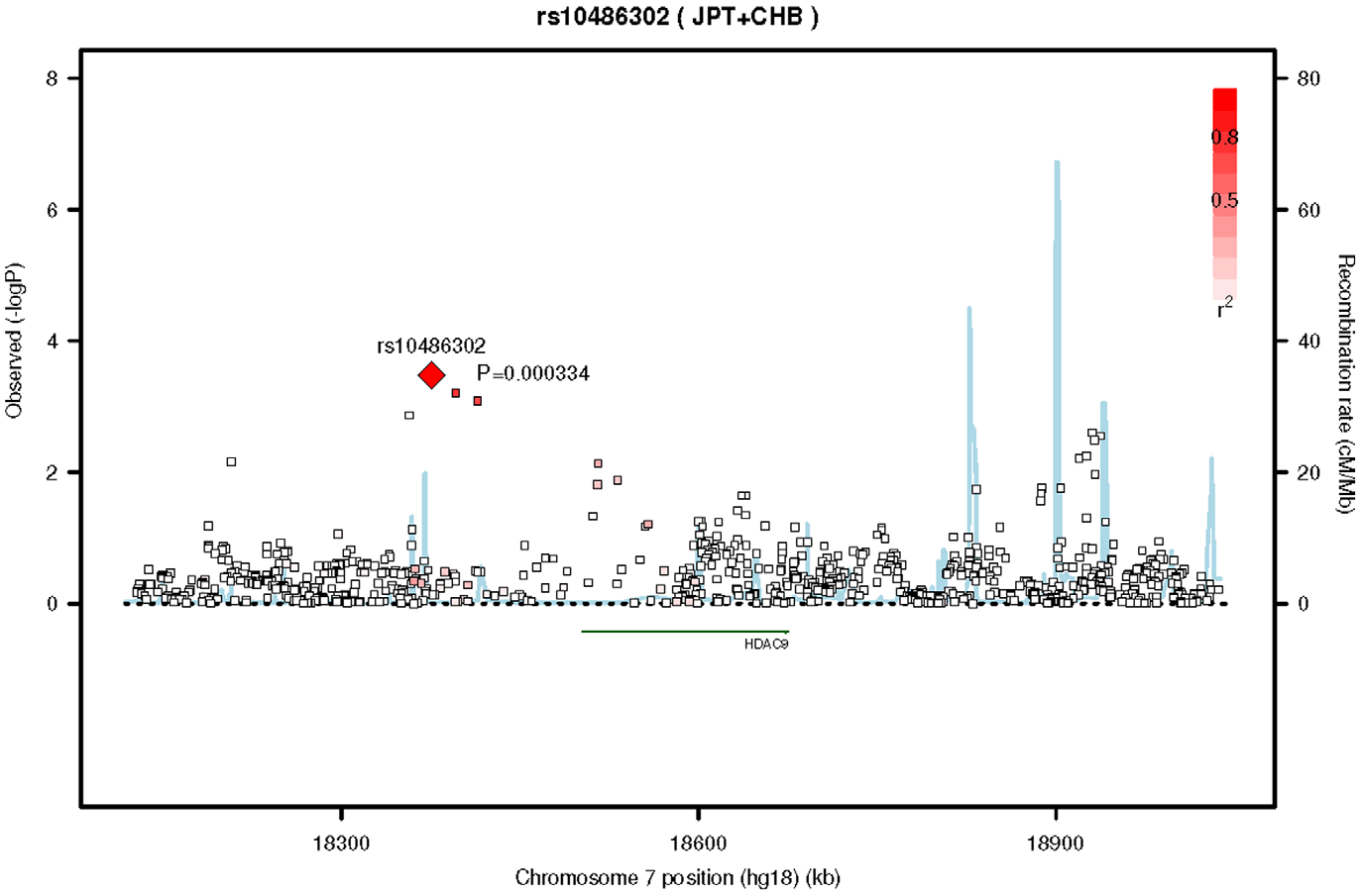
Regional association plot of SNP rs10486302 on chromosome 7 for Singapore Asian Indians. This figure is the regional association plot of SNP rs10486302 on chromosome 7 that reached gene-wide significance (p<4.9×10^−4^) in the testing of transferability of the discovery SNP, rs12155400, to other SNPs in the histone deacetylase 9 gene in a cohort of Singapore Asian Indians without diabetes or hypertension. The lead and surrounding SNPs are color coded according to the pair-wise linkage disequilibrium (LD) with the lead SNP (presented as a diamond) on a scale of r^2^ from 0 to 1. Estimated recombination rates reflect the local LD structure in the 500kb buffer around the index SNP and plotted based on values from HapMap II Han Chinese of Beijing China (HCT)/Japanese in Tokyo, Japan (JPT) populations.

**Figure 4:**
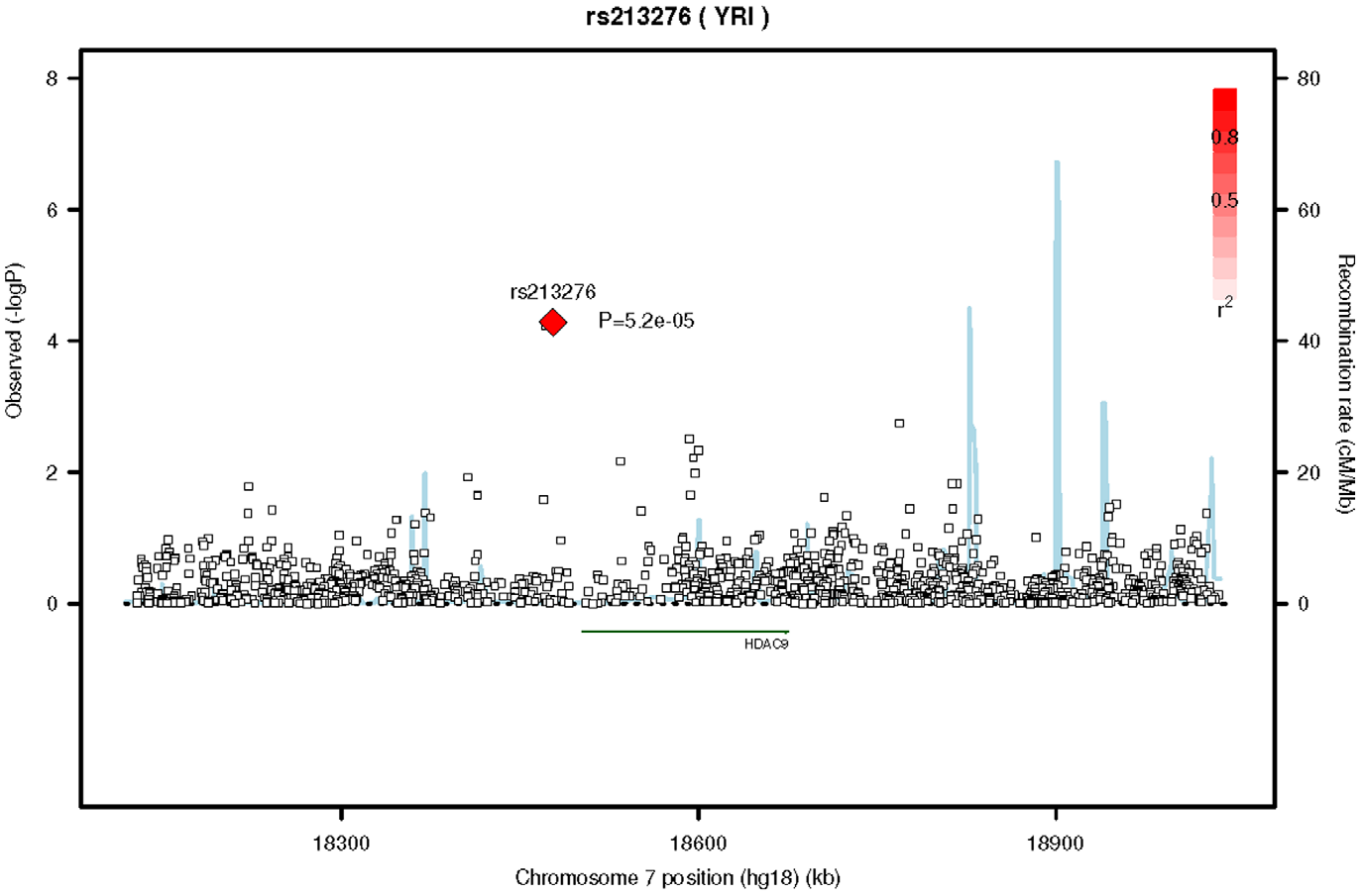
Regional association plot of SNP rs213276 on chromosome 7 for African Americans. This figure is the regional association plot of SNP rs213276 on chromosome 7 that reached gene-wide significance (p<5.2×10^−5^) in the testing of transferability of the discovery SNP, rs12155400, to other SNPs in the histone deacetylase 9 gene in a cohort of African Americans without diabetes or hypertension. The lead and surrounding SNPs are color coded according to the pair-wise linkage disequilibrium (LD) with the lead SNP (presented as a diamond) on a scale of r^2^ from 0 to 1. Estimated recombination rates reflect the local LD structure in the 500 kb buffer around the index SNP and plotted based on values from HapMap II Yoruba in Ibadan, Nigeria (YRI) population.

We tested the transferability of this SNP and locus (other SNPs in the *HDAC9* gene) in a cohort of Singapore Asian Indians (SINDI) and a cohort of African Americans in MESA. In both cases these cohorts were restricted to participants without diabetes or hypertension. The SNP, rs12155400, did not extend to either the Singapore Asian Indians (beta = 1.1±1.23, p = 0.39, MAF = 0.01, n = 1,694) or the African Americans (beta = 0.7±1.40, p = 0.63, n = 492, MAF = 0.01). However, the power for these studies was around 60% for the Singapore Asian Indians and less than 10% for the African Americans, Figure S7. One SNP did reach gene-wide significance (p<4.9×10^−4^) in the Singapore Asian Indians, at SNP rs10486302 (p = 3.3×10^−4^), Figure 3. In addition, two other SNPs at this locus in linkage disequilibrium with rs10486302 just missed reaching gene-wide significance, rs723296 (p = 6.2×10^−4^, r^2^ = 0.79) an imputed SNP and rs12374816 (p = 8.2×10^−4^, r^2^ = 0.72) a genotyped SNP. SNP rs12155400 and rs10486302 are not in linkage disequilibrium but they do lie within the confines of the same recombination hotspots. In African Americans, a locus of 3 imputed SNPs reached gene-wide significance (p<1.7×10^−4^) around SNP rs213276 (p = 5.2×10^−5^), Figure 4. The two other SNPs in linkage disequilibrium included rs213274 (p = 5.5×10^−5^) and rs213273 (p = 5.8×10^−5^). These SNPs lie in the zone adjacent to the recombination hotspots shared by the other two ethnic groups.

In silico look-ups of the association between SNP rs12155400 and several micro- and macrovascular diseases were done and the results are shown in Table 3. In participants without hypertension in the CKDGen consortium, the SNP was marginally associated with microalbuminuria and chronic kidney disease (p = 0.02 and 0.04 respectively) before correcting for multiple testing but was not associated with the urinary albumin/creatinine ratio or renal function estimated by serum creatinine. The small minor allele frequency prevented testing in the Heart and Vascular Health Study. There was no association between this SNP and coronary artery disease in the Wellcome Trust Case Control Consortium or with anemia in the CHARGE Hematology Working Group.

**Table 3:** The association between SNP rs12155400 on chromosome 7 and various micro- and macrovascular diseases.

Cohort	n	MAF	Beta	SE	p-value
WTCCC*					
Coronary Artery Disease	4,864	0.028	0.05	0.15	0.75
HVH†					
Stroke	1,815	0.009	#	#	#
Myocardial Infarction	2,486	0.009	#	#	#
CKDGen Consortium‡					
All participants					
Microalbuminuria	31,580	0.021	−0.18	0.12	0.12
Chronic Kidney Disease	62,237	0.023	−0.15	0.09	0.11
UACR	31,580	0.020	−0.03	0.04	0.51
eGFRcrea	67,093	0.022	0.00	0.01	0.66
Participants without hypertension					
Microalbuminuria	18,501	0.021	−0.39	0.16	0.02
Chronic Kidney Disease	36,649	0.021	−0.26	0.13	0.04
UACR	19,447	0.020	−0.04	0.04	0.33
eGFRcrea	40,759	0.023	0.00	0.01	0.64
Participants without diabetes					
Microalbuminuria	25,914	0.021	−0.25	0.13	0.06
Chronic Kidney Disease	35,107	0.021	−0.14	0.13	0.25
UACR	26,652	0.020	−0.03	0.04	0.44
eGFRcrea	39,125	0.021	0.00	0.01	0.94
CHARGE Hematology Working Group					
Anemia	25,192	0.019	−0.23	0.16	0.14

* The Wellcome Trust Case Control Consortium 2.

† The Heart and Vascular Health Study.

# No results.

‡ Chronic Kidney Diseases Genetics Consortium. MAF: Minor Allele Frequency. SE: Standard Error. UACR: Urinary Albumin to Creatinine Ratio. eGFRcrea: estimated Glomerular Filtration Rate using creatinine.

We examined our findings in a set of SNPs that have previously been shown to be associated with diabetes or hypertension in other GWAS studies and two SNPs associated with diabetic retinopathy in a recently published candidate gene study. None of these SNPs were associated with retinopathy (p<4.8×10^−4^) in the primary analytical group or either subgroup stratified by hypertension status, Tables S4, S5 and S6.

## Discussion

Results of our primary analysis and secondary analysis of individuals with hypertension showed no evidence of a genetic association with mild retinopathy. One SNP, rs12155400, reached genome-wide significant association with mild retinopathy in individuals of European ancestry without diabetes and hypertension, in the histone deacetylase 9 gene (*HDAC9*) gene on chromosome 7. The available evidence suggests this is a false positive finding. The SNP is located in an intronic region. The minor allele frequency was low, less than 2%. The SNP was imputed and the quality of the imputation was modest at best with an r^2^ of about 0.7. There were no other common variants in the *HDAC9* gene associated with the outcome. The SNP findings did not extend to a cohort of Singapore Asian Indians or African Americans although there was limited power to detect an association in these smaller cohorts.

Diabetes and hypertension are the primary risk factors for retinopathy [22], [23] but the etiology of mild retinopathy in individuals without diabetes remains unclear [4]. Determining these risk factors may shed light on the association between mild retinopathy and other conditions such as renal dysfunction [10], [11], [12], incident hypertension [1], clinical stroke [13], [14], [15], [16], [17], congestive heart failure [18], cardiovascular mortality [19], [20] and risk of clinical diabetes [21].

Common genetic factors are thought to underlie the pathogenesis of microvascular disease in the eye and kidney, particularly in persons without the major risk factors for pathologies in these organs; diabetes and hypertension. There are congenital syndromes associated with genetic defects affecting both the retinal and renal vasculature such as von Hippel Lindau Disease and Sturge-Weber-Krabble Syndrome [48].

We found a number of SNPs in our study with highly suggestive p-values (p<9.99E−06) some of which are associated with retinal degenerations. For example, in the primary analysis there were two SNPs associated with mild retinopathy, rs7553035 and rs21661074 near *RD3* on chromosome 1. This gene encodes a retinal protein that is associated with promyelocytic leukemia-gene product bodies in the nucleus. Mutations in this gene cause Leber’s congenital amaurosis type 12 [49]. Another locus of interest in the secondary analysis of subjects with hypertension included SNPs near *TEAD1* on chromosome 11, a gene associated with peripapillary chorioretinal degeneration [50]. In this same group, SNP rs1933752 near the *UTRN* gene on chromosome 6 was also associated mild retinopathy. The protein encoded by this gene shares both structural and functional similarities with the dystrophin gene. Dystrophins are normally part of critical cytoskeleton-associated membrane-bound molecular scaffolds involved in blood-brain barrier function [51].

The negative results from our study do not completely rule out genetic effects in the development of mild retinopathy. Limitations in our study make finding these associations difficult. The combined phenotype of microaneurysms or dot-blot hemorrhages is transitory in nature [2]. Photos in each of these studies were taken with a limited number of fields and sometimes only in one eye. There are potentially many pathways to develop both dot-blot hemorrhages and microaneurysms some of which may be unique to each phenotype. All of these limitations lead to misclassifications that tend to bias results towards the null.

Despite the lack of genome-wide significant findings, this study does represent the first large scale genetic study of mild retinopathy in a population based cohort. This work serves to highlight some of the difficulties in studying this phenotype particularly using cross sectional retinal observations. Hopefully it will provide insights for better research methods in the future as well as serve as a resource for candidate gene analyses of some of our highly suggestive hits in biologically relevant genes or rs12155400 in *HDAC9. HDAC9* has recently been reported to be associated with large vessel ischemic stroke [52] and there was evidence of locus transferability to other SNPs in *HDAC9* that reached gene-wide significance in Asians and African Americans.

In conclusion, results from this study showed little evidence that the presence of mild retinopathy in individuals without diabetes was associated with SNPs. Further studies are needed to explore the remaining highly suggestive SNPs. This may include fine mapping in *HDAC9*, gene-environment interaction studies or pathway analyses. Identifying genes associated with these signs may help unravel novel pathways and determinants of microvascular diseases.

## Supporting Information

Figure S1 **QQ-plot from primary analysis including all individuals without diabetes.**
(TIFF)

Figure S2 **Manhattan plot from primary analysis including all individuals without diabetes.**
(TIF)

Figure S3 **QQ-plot from primary analysis including all individuals without diabetes that have hypertension.**
(TIFF)

Figure S4 **Manhattan plot from primary analysis including all individuals without diabetes that have hypertension.**
(TIF)

Figure S5 **QQ-plot from primary analysis including all individuals without diabetes or hypertension.**
(TIFF)

Figure S6 **Manhattan plot from primary analysis including all individuals without diabetes or hypertension.**
(TIF)

Figure S7 **Power calculations for replication of a SNP with a beta = −1.33 and allele frequency = 0.98 for multiple prevalence levels of retinopathy.**
(TIF)

Table S1 **Highly suggestive hits (p<9.99E−06) from the primary GWAS of individuals without diabetes plus genome-wide significant SNP, rs12155400 on chromosome 7 from the secondary GWAS of individuals without hypertension.**
(DOCX)

Table S2 **Highly suggestive hits (p<9.99E−06) from the secondary GWAS of individuals with hypertension plus genome-wide significant SNP, rs12155400 on chromosome 7 from the secondary GWAS of individuals without hypertension.**
(DOCX)

Table S3 **Highly suggestive hits (p<9.99E−06) from the secondary GWAS of individuals without hypertension.**
(DOCX)

Table S4 **Meta-analysis results in SNPs associated with diabetes.**
(DOCX)

Table S5 **Meta-analysis results in SNPs associated with SBP, DBP and HTN.**
(DOCX)

Table S6 **Meta-analysis results in SNPs associated with diabetic retinopathy.**
(DOCX)
