# Supplementary figures and images for: Genome-Wide Association Study of Retinopathy in Individuals without Diabetes
Source: PLoS One. 2013 Feb 5;8(2):e54232. doi: 10.1371/journal.pone.0054232 (PMC3564946; doi:10.1371/journal.pone.0054232)

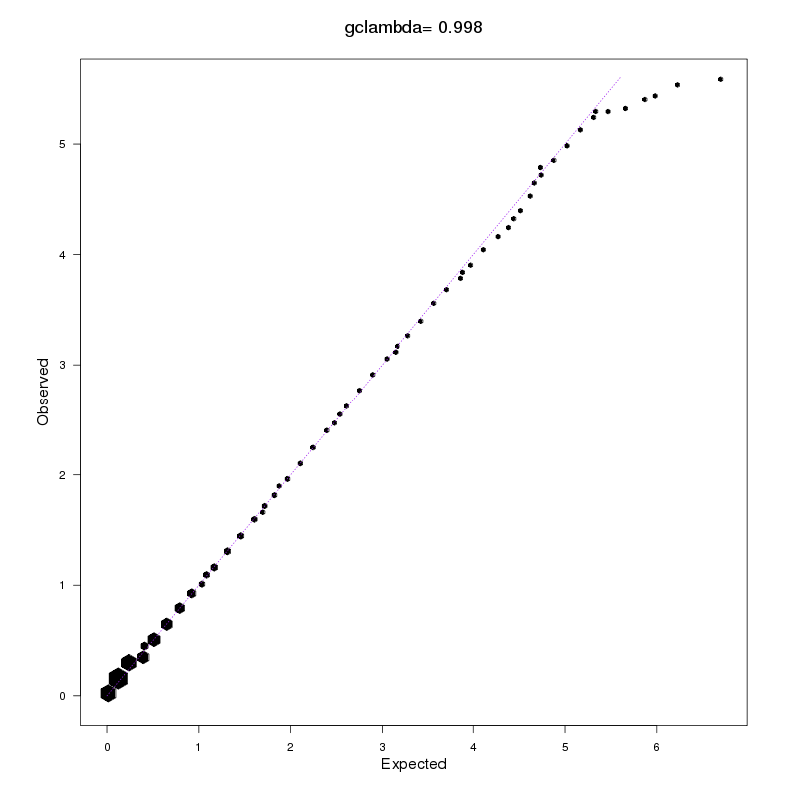

Supplement: Figure S1 — QQ-plot from primary analysis including all individuals without diabetes. (TIFF) [file pone.0054232.s001.tif]

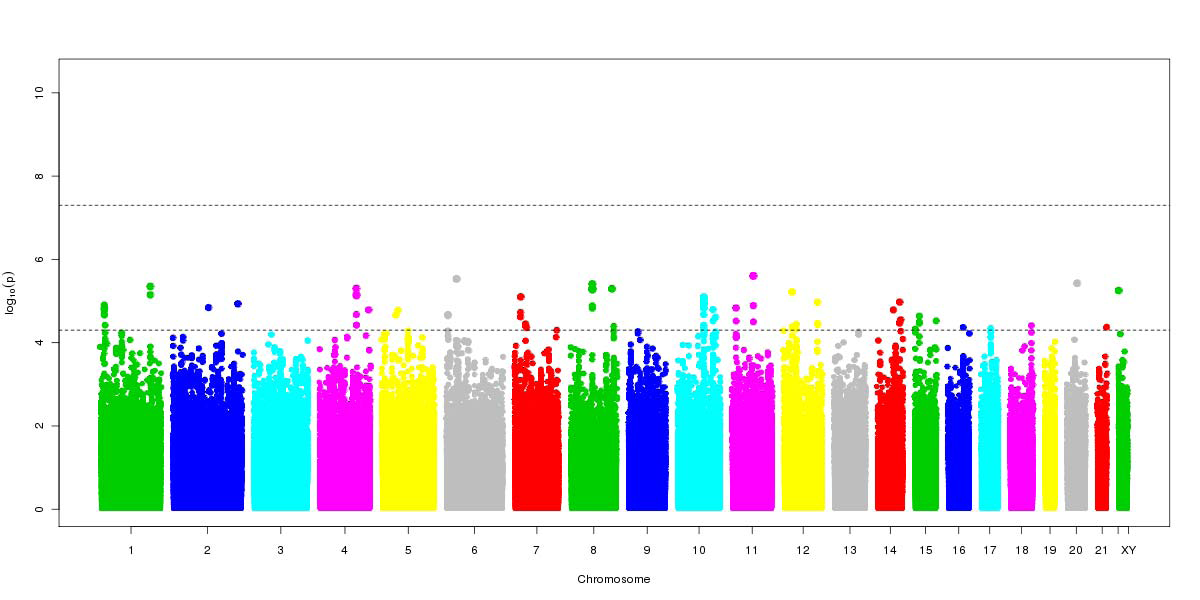

Supplement: Figure S2 — Manhattan plot from primary analysis including all individuals without diabetes. (TIF) [file pone.0054232.s002.tif]

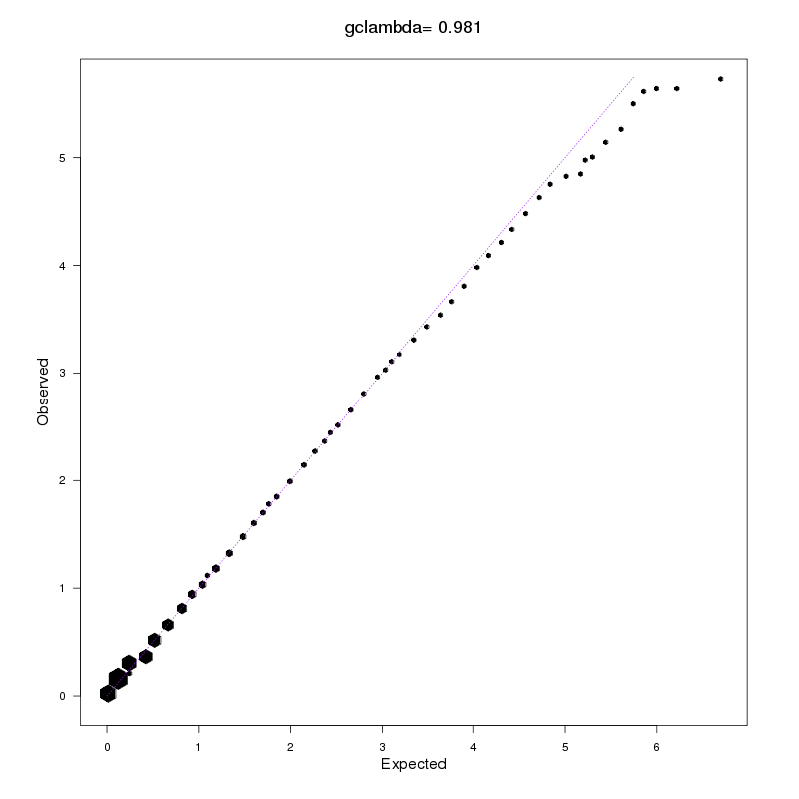

Supplement: Figure S3 — QQ-plot from primary analysis including all individuals without diabetes that have hypertension. (TIFF) [file pone.0054232.s003.tif]

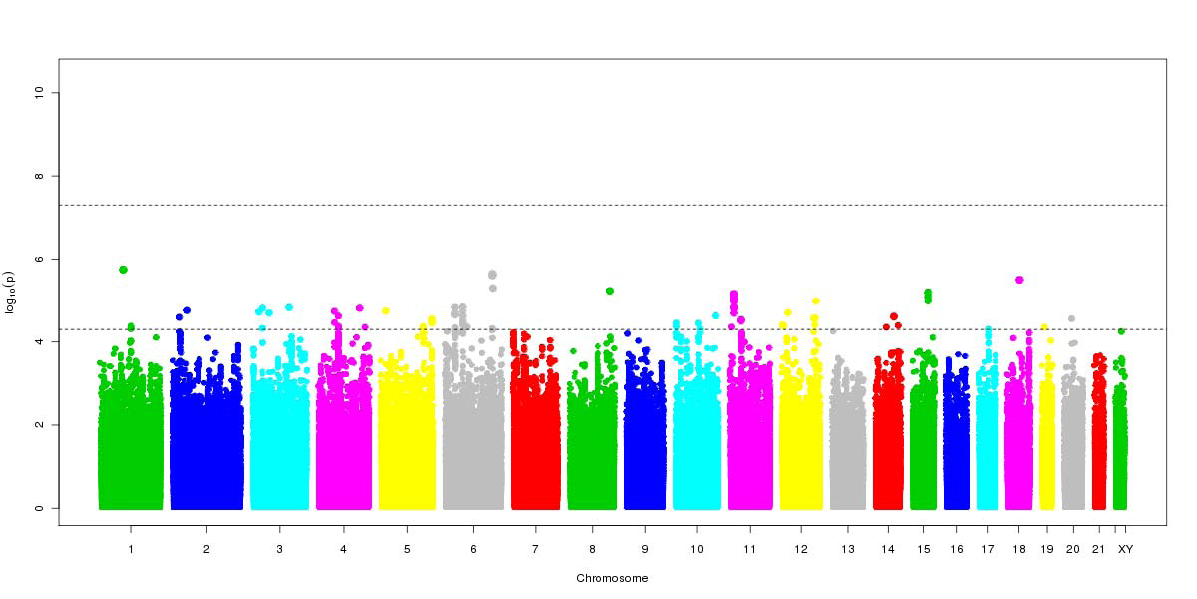

Supplement: Figure S4 — Manhattan plot from primary analysis including all individuals without diabetes that have hypertension. (TIF) [file pone.0054232.s004.tif]

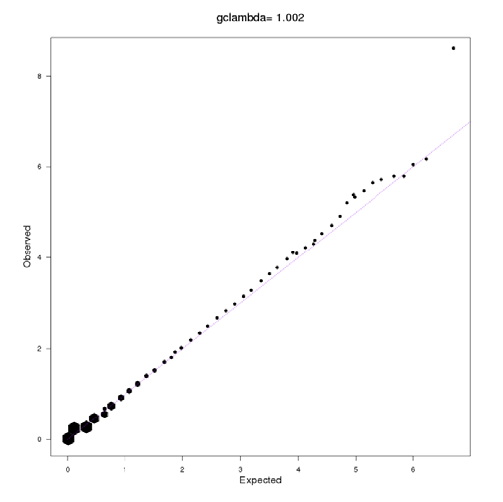

Supplement: Figure S5 — QQ-plot from primary analysis including all individuals without diabetes or hypertension. (TIFF) [file pone.0054232.s005.tif]

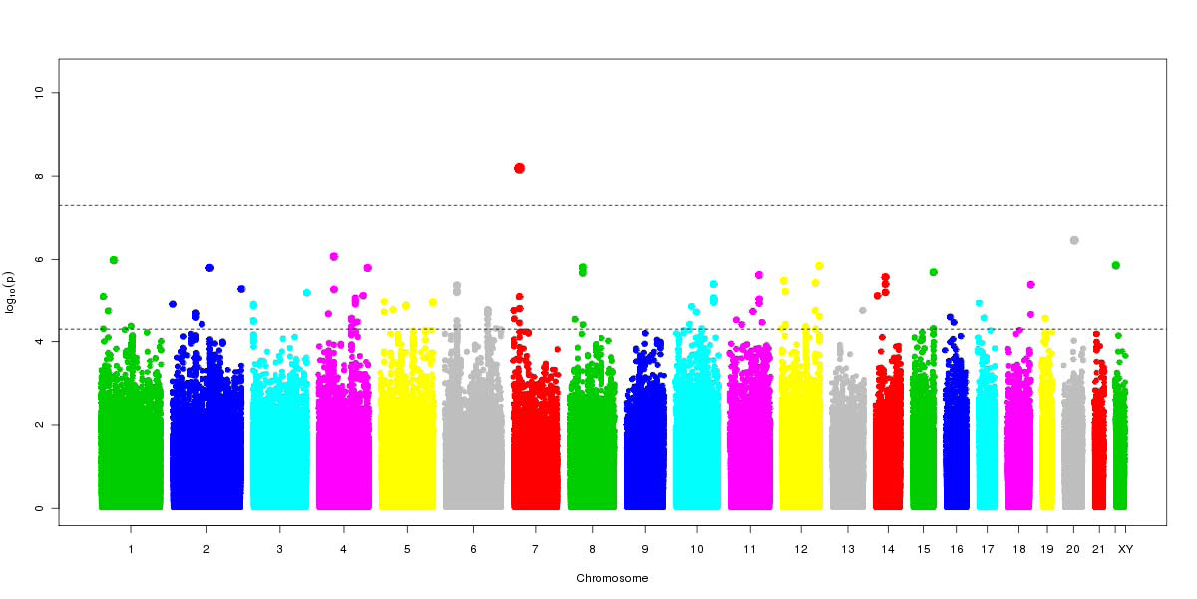

Supplement: Figure S6 — Manhattan plot from primary analysis including all individuals without diabetes or hypertension. (TIF) [file pone.0054232.s006.tif]

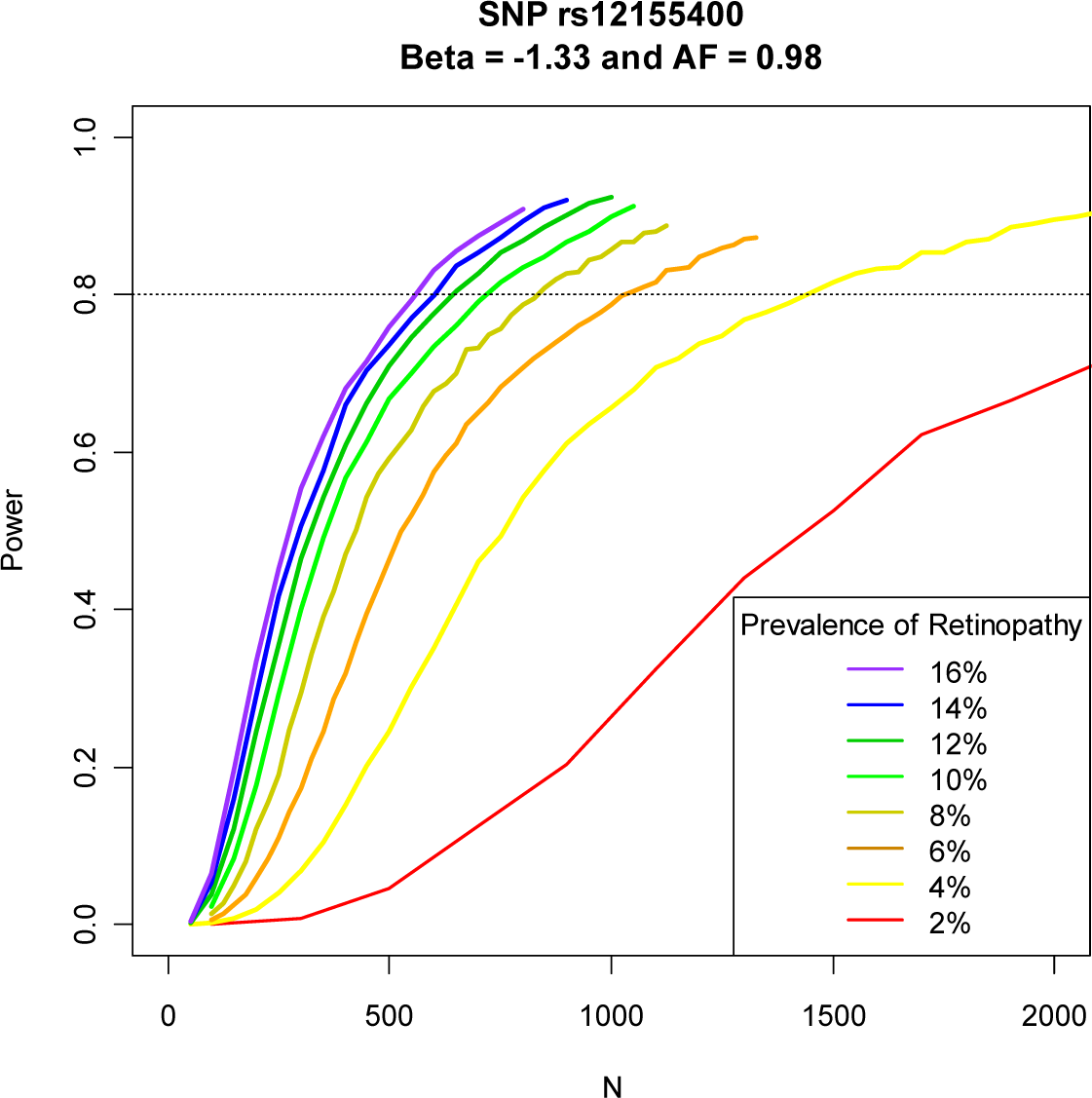

Supplement: Figure S7 — Power calculations for replication of a SNP with a beta = −1.33 and allele frequency = 0.98 for multiple prevalence levels of retinopathy. (TIF) [file pone.0054232.s007.tif]
