# Supplementary material for: Genome-Wide Association Study of Retinopathy in Individuals without Diabetes
Source: PLoS One. 2013 Feb 5;8(2):e54232. doi: 10.1371/journal.pone.0054232 (PMC3564946; doi:10.1371/journal.pone.0054232)
Supplement: Table S1 — Highly suggestive hits (p<9.99E−06) from the primary GWAS of individuals without diabetes plus genome-wide significant SNP, rs12155400 on chromosome 7 from the secondary GWAS of individuals without hypertension. (DOCX) [file pone.0054232.s008.docx]

| Table S1, highly suggestive hits (p < 9.99E-06) from the primary GWAS of individuals without diabetes plus genome-wide significant SNP, rs12155400 on chromosome 7 from the secondary GWAS of individuals without hypertension. | | | | | | | | | | |
| --- | --- | --- | --- | --- | --- | --- | --- | --- | --- | --- |
| SNPID | Chr | Position | ClosestRefGene | N | A1 | F1 | beta | SE | p | Direction* |
| rs2166104 | 1 | 209757441 | RD3 | 19411 | a | 0.92 | -0.35 | 0.08 | 7.13E-06 | +---+- |
| rs7553035 | 1 | 209758329 | RD3 | 19411 | t | 0.07 | 0.37 | 0.08 | 4.49E-06 | -+++-+ |
| rs10001004 | 4 | 138348249 | PCDH18 | 19411 | a | 0.02 | 0.56 | 0.12 | 6.88E-06 | -++--+ |
| rs7692267 | 4 | 138349472 | PCDH18 | 19411 | t | 0.98 | -0.56 | 0.12 | 7.33E-06 | +--++- |
| rs7666605 | 4 | 138354981 | PCDH18 | 19411 | a | 0.98 | -0.56 | 0.12 | 7.39E-06 | +--++- |
| rs10004839 | 4 | 138374262 | PCDH18 | 19411 | t | 0.02 | 0.57 | 0.13 | 5.03E-06 | -++--+ |
| rs9378134 | 6 | 29459763 | OR12D3 | 19411 | a | 0.04 | 0.49 | 0.1 | 2.94E-06 | ++-+++ |
| rs12155400 | 7 | 18395446 | HDAC9 | 18445 | a | 0.98 | -0.73 | 0.16 | 7.95E-06 | ?--+-- |
| rs7827287 | 8 | 65768273 | CYP7B1^†^ | 19411 | t | 0.51 | 0.2 | 0.04 | 4.97E-06 | ++++++ |
| rs10957321 | 8 | 65768432 | CYP7B1^†^ | 19411 | a | 0.51 | 0.2 | 0.04 | 5.12E-06 | ++++++ |
| rs7830315 | 8 | 65770915 | CYP7B1^†^ | 19411 | t | 0.49 | -0.2 | 0.04 | 5.32E-06 | ------ |
| rs10112206 | 8 | 65774315 | CYP7B1^†^ | 19411 | a | 0.48 | -0.2 | 0.04 | 4.90E-06 | ------ |
| rs6472147 | 8 | 65775251 | CYP7B1^†^ | 19411 | a | 0.48 | -0.2 | 0.04 | 5.32E-06 | ------ |
| rs6472155 | 8 | 65892761 | CYP7B1 | 19411 | a | 0.51 | -0.23 | 0.05 | 3.90E-06 | ------ |
| rs9918807 | 8 | 130745821 | MLZE | 19411 | t | 0.94 | -0.47 | 0.1 | 5.10E-06 | ------ |
| rs1329202 | 10 | 83178748 | NRG3 | 19411 | t | 0.66 | 0.21 | 0.05 | 9.12E-06 | ++++++ |
| rs1329201 | 10 | 83178933 | NRG3 | 19411 | t | 0.34 | -0.22 | 0.05 | 8.07E-06 | ------ |
| rs12260387 | 10 | 83181721 | NRG3 | 19411 | a | 0.66 | 0.22 | 0.05 | 8.56E-06 | ++++++ |
| rs11826937 | 11 | 78336793 | ODZ4 | 19411 | a | 0.97 | -0.6 | 0.13 | 2.49E-06 | ---+-- |
| rs258401 | 12 | 27900036 | KLHDC5 | 19411 | t | 0.26 | 0.24 | 0.05 | 6.08E-06 | -+++++ |
| rs17194885 | 20 | 35501803 | SRC | 19411 | a | 0.96 | -0.61 | 0.13 | 3.73E-06 | +----- |
| rs5763911 | 22 | 19127289 | KLHL22^†^ | 18445 | t | 0.96 | -0.52 | 0.11 | 5.60E-06 | ?----- |
| *Order: CHS, AGES, ARIC, BMES, MESA, RS | | | |  |  |  |  |  |  |  |
| ^†^In the gene |  |  |  |  |  |  |  |  |  |  |
