# Supplementary material for: Genome-Wide Association Study of Retinopathy in Individuals without Diabetes
Source: PLoS One. 2013 Feb 5;8(2):e54232. doi: 10.1371/journal.pone.0054232 (PMC3564946; doi:10.1371/journal.pone.0054232)
Supplement: Table S2 — Highly suggestive hits (p<9.99E−06) from the secondary GWAS of individuals with hypertension plus genome-wide significant SNP, rs12155400 on chromosome 7 from the secondary GWAS of individuals without hypertension. (DOCX) [file pone.0054232.s009.docx]

| Table S2, highly suggestive hits (p < 9.99E-06) from the secondary GWAS of individuals with hypertension plus genome-wide significant SNP, rs12155400 on chromosome 7 from the secondary GWAS of individuals without hypertension. | | | | | | | | | | |
| --- | --- | --- | --- | --- | --- | --- | --- | --- | --- | --- |
| SNPID | Chr | Position | ClosestRefGene | N | A1 | F1 | beta | se | p | Direction* |
| rs17130538 | 1 | 88852132 | PKN2 | 7645 | t | 0.01 | 1.32 | 0.28 | 1.79E-06 | ??++?- |
| rs3761997 | 6 | 143526480 | AIG1^†^ | 8867 | t | 0.22 | 0.31 | 0.07 | 2.48E-06 | ++++++ |
| rs2154404 | 6 | 143528970 | AIG1^†^ | 8867 | t | 0.78 | -0.31 | 0.07 | 2.31E-06 | ------ |
| rs4896622 | 6 | 143530528 | AIG1^†^ | 8867 | a | 0.78 | -0.32 | 0.07 | 2.29E-06 | ------ |
| rs1933752 | 6 | 145226962 | UTRN | 8867 | t | 0.74 | -0.29 | 0.06 | 5.00E-06 | ------ |
| rs12155400 | 7 | 18395446 | HDAC9 | 8373 | a | 0.98 | -0.25 | 0.23 | 0.28 | ?----- |
| rs9918807 | 8 | 130745821 | MLZE | 8867 | t | 0.94 | -0.58 | 0.13 | 5.82E-06 | ------ |
| rs12295437 | 11 | 12950915 | TEAD1 | 8373 | t | 0.95 | -0.55 | 0.12 | 9.20E-06 | ?----- |
| rs11022581 | 11 | 12951988 | TEAD1 | 8373 | t | 0.16 | 0.38 | 0.08 | 6.80E-06 | ?+-+++ |
| rs7102071 | 11 | 12970664 | LOC644943 | 8373 | a | 0.95 | -0.53 | 0.12 | 7.97E-06 | ?----- |
| rs4353448 | 15 | 78945127 | KIAA1199^†^ | 8867 | a | 0.42 | 0.27 | 0.06 | 6.32E-06 | ++++++ |
| rs4145805 | 15 | 78947317 | KIAA1199^†^ | 8867 | a | 0.42 | 0.26 | 0.06 | 8.11E-06 | ++++++ |
| rs4939560 | 18 | 44539143 | KIAA0427^†^ | 8867 | a | 0.34 | 0.28 | 0.06 | 3.16E-06 | ++++++ |
| *Order: CHS, AGES, ARIC, BMES, MESA, RS | | | |  |  |  |  |  |  |  |
| ^†^ In reference gene | | |  |  |  |  |  |  |  |  |
