# Supplementary material for: Genome-Wide Association Study of Retinopathy in Individuals without Diabetes
Source: PLoS One. 2013 Feb 5;8(2):e54232. doi: 10.1371/journal.pone.0054232 (PMC3564946; doi:10.1371/journal.pone.0054232)
Supplement: Table S3 — Highly suggestive hits (p<9.99E−06) from the secondary GWAS of individuals without hypertension. (DOCX) [file pone.0054232.s010.docx]

| Table S3, highly suggestive hits (p < 9.99E-06) from the secondary GWAS of individuals without hypertension. | | | | | | | | | | |
| --- | --- | --- | --- | --- | --- | --- | --- | --- | --- | --- |
| SNPID | Chr | Position | ClosestRefGene | N | A1 | F1 | beta | se | p | Direction* |
| rs12029721 | 1 | 15089076 | KIAA1026^†^ | 10522 | a | 0.93 | -0.74 | 0.17 | 7.91E-06 | ------ |
| rs17117148 | 1 | 58466331 | DAB1^†^ | 5012 | c | 0.01 | 2.48 | 0.51 | 1.05E-06 | ?++??? |
| rs4662573 | 2 | 127658268 | CYP27C1^†^ | 10522 | t | 0.53 | 0.35 | 0.07 | 1.60E-06 | ++++++ |
| rs4854078 | 2 | 240831417 | OTOS | 10050 | a | 0.02 | 0.98 | 0.22 | 5.15E-06 | ?-++++ |
| rs6771223 | 3 | 195152130 | HES1 | 8633 | a | 0.27 | 0.79 | 0.18 | 6.40E-06 | +++??+ |
| rs7696751 | 4 | 57730614 | IGFBP7 | 10522 | c | 0.96 | -0.68 | 0.15 | 5.29E-06 | ---+-- |
| rs10488927 | 4 | 57753542 | IGFBP7 | 10522 | t | 0.05 | 0.7 | 0.14 | 8.62E-07 | +++-++ |
| rs10001004 | 4 | 138348249 | PCDH18 | 10522 | a | 0.03 | 0.78 | 0.18 | 9.83E-06 | -++--+ |
| rs10004839 | 4 | 138374262 | PCDH18 | 10522 | t | 0.03 | 0.8 | 0.18 | 8.67E-06 | --+--+ |
| rs7656265 | 4 | 168871755 | ANXA10 | 6320 | t | 0.01 | 1.8 | 0.4 | 7.48E-06 | ?-+?+? |
| rs10027571 | 4 | 183598930 | ODZ3^†^ | 5012 | t | 0.01 | 2.49 | 0.52 | 1.60E-06 | ??+??? |
| rs9264733 | 6 | 31352193 | HLA-C | 10522 | c | 0.27 | 0.35 | 0.08 | 4.21E-06 | ++++++ |
| rs9264740 | 6 | 31352310 | HLA-C | 10522 | t | 0.73 | -0.35 | 0.08 | 4.21E-06 | ------ |
| rs6919908 | 6 | 31352939 | HLA-C | 10522 | t | 0.32 | 0.33 | 0.07 | 5.67E-06 | ++++++ |
| rs6906846 | 6 | 31353715 | HLA-C | 10522 | a | 0.31 | 0.33 | 0.07 | 6.23E-06 | ++++++ |
| rs12536864 | 7 | 18185106 | PRPS1L1 | 10050 | a | 0.97 | -0.81 | 0.18 | 7.91E-06 | ?--+-- |
| rs12155400 | 7 | 18395446 | HDAC9^†^ | 10050 | a | 0.98 | -1.33 | 0.23 | 6.55E-09 | ?--+-- |
| rs7001440 | 8 | 31120402 | WRN^†^ | 10522 | a | 0.07 | -0.73 | 0.15 | 2.11E-06 | ------ |
| rs6988789 | 8 | 31129830 | WRN^†^ | 10522 | t | 0.07 | -0.74 | 0.15 | 1.56E-06 | ------ |
| rs1248077 | 10 | 121129035 | GRK5* | 10522 | a | 0.95 | -0.64 | 0.14 | 3.97E-06 | -+-+-- |
| rs4751714 | 10 | 121130501 | GRK5^†^ | 10522 | a | 0.04 | 0.64 | 0.14 | 9.16E-06 | +-+-++ |
| rs2275037 | 10 | 121130524 | GRK5^†^ | 10522 | c | 0.05 | 0.62 | 0.14 | 9.82E-06 | +-+-++ |
| rs3818779 | 10 | 121130661 | GRK5^†^ | 10522 | a | 0.05 | 0.62 | 0.14 | 9.89E-06 | +-+-++ |
| rs11813472 | 10 | 121136889 | GRK5^†^ | 10522 | a | 0.04 | 0.71 | 0.16 | 8.54E-06 | +-+-++ |
| rs10895016 | 11 | 100176135 | TMEM133 | 10522 | a | 0.05 | 0.67 | 0.14 | 2.38E-06 | +-++++ |
| rs11605920 | 11 | 100199520 | TMEM133 | 10522 | t | 0.95 | -0.65 | 0.15 | 9.23E-06 | -+---- |
| rs10895018 | 11 | 100201327 | TMEM133 | 10522 | a | 0.95 | -0.66 | 0.15 | 9.09E-06 | -+---- |
| rs7957898 | 12 | 10305627 | GABARAPL1 | 5012 | a | 0.99 | -3.74 | 0.81 | 3.31E-06 | ?--??? |
| rs7304327 | 12 | 14038615 | GRIN2B | 10522 | a | 0.05 | 0.62 | 0.14 | 5.98E-06 | ++++++ |
| rs12580480 | 12 | 114448256 | MED13L | 10050 | a | 0.98 | -1.01 | 0.22 | 3.71E-06 | ??---- |
| rs7310361 | 12 | 128161242 | TMEM132D^†^ | 10522 | t | 0.05 | 0.82 | 0.17 | 1.46E-06 | ++++++ |
| rs2022878 | 14 | 27114667 | NOVA1 | 8161 | a | 0.99 | -2.54 | 0.57 | 7.53E-06 | ?--??- |
| rs6572930 | 14 | 53509154 | BMP4 | 10522 | t | 0.03 | 0.98 | 0.22 | 6.20E-06 | -++-++ |
| rs942316 | 14 | 53510733 | BMP4 | 10050 | a | 0.03 | 0.96 | 0.21 | 3.92E-06 | ?++-++ |
| rs4316669 | 14 | 53512418 | BMP4 | 10050 | t | 0.97 | -1.04 | 0.22 | 2.67E-06 | ?--+-- |
| rs10902540 | 15 | 96498855 | ARRDC4 | 10522 | t | 0.6 | -0.34 | 0.07 | 2.03E-06 | ------ |
| rs7233024 | 18 | 75426434 | NFATC1 | 10522 | a | 0.11 | 0.55 | 0.12 | 4.09E-06 | -+++++ |
| rs17194885 | 20 | 35501803 | SRC | 10050 | a | 0.96 | -0.97 | 0.19 | 3.49E-07 | ?----- |
| rs5763911 | 22 | 19127289 | KLHL22^†^ | 10050 | t | 0.96 | -0.87 | 0.18 | 1.40E-06 | ?-+--- |
| *Direction order: CHS, AGES, ARIC, BMES, MESA, RS. | | | |  |  |  |  |  |  |  |
| ^†^ In reference gene. | | |  |  |  |  |  |  |  |  |
