# Supplementary material for: Genome-Wide Association Study of Retinopathy in Individuals without Diabetes
Source: PLoS One. 2013 Feb 5;8(2):e54232. doi: 10.1371/journal.pone.0054232 (PMC3564946; doi:10.1371/journal.pone.0054232)
Supplement: Table S4 — Meta-analysis results in SNPs associated with diabetes. (DOCX) [file pone.0054232.s011.docx]

| Table S4, association between known diabetes SNPs and retinopathy in subjects without diabetes. | | | | | | | | | | | | |  |  |  |  |  |
| --- | --- | --- | --- | --- | --- | --- | --- | --- | --- | --- | --- | --- | --- | --- | --- | --- | --- |
|  |  |  | All Subjects | | | | | Subjects with Hypertension | | | | | Subjects without Hypertension | | | | |
| SNP | Chr | Locus | beta | se | p | n | direction | beta | se | p | n | direction | beta | se | p | n | Direction* |
| rs10923931 | 1 | NOTCH2 | -0.09 | 0.07 | 0.22 | 19411 | --+--- | -0.13 | 0.09 | 0.17 | 8867 | +-+--- | -0.04 | 0.11 | 0.75 | 10522 | -+---- |
| rs340874 | 1 | PROX1 | -0.03 | 0.05 | 0.45 | 19411 | ----+- | 0.00 | 0.06 | 0.99 | 8867 | +-+-+- | -0.08 | 0.07 | 0.28 | 10522 | -+--+- |
| rs2943641 | 2 | IRS1 | 0.08 | 0.05 | 0.08 | 19411 | --++++ | 0.12 | 0.06 | 0.04 | 8867 | +-++++ | 0.03 | 0.07 | 0.71 | 10522 | ---+-+ |
| rs7578597 | 2 | THADA | -0.02 | 0.07 | 0.81 | 19411 | +-+--+ | -0.11 | 0.09 | 0.25 | 8867 | +-+--- | 0.06 | 0.11 | 0.60 | 10050 | ?-++++ |
| rs7593730 | 2 | RBMS1/ITGB6 | -0.02 | 0.05 | 0.73 | 19411 | -+++-- | 0.06 | 0.07 | 0.42 | 8867 | -+++-- | -0.12 | 0.09 | 0.15 | 10522 | -+---- |
| rs243021 | 2 | BCL11A | 0.01 | 0.05 | 0.82 | 19411 | +++--+ | -0.05 | 0.06 | 0.37 | 8867 | ----+- | 0.10 | 0.07 | 0.17 | 10522 | ++++-+ |
| rs780094 | 2 | GCKR | -0.05 | 0.05 | 0.32 | 19411 | -++--- | 0.00 | 0.06 | 0.94 | 8867 | +++--+ | -0.10 | 0.07 | 0.18 | 10522 | -++-+- |
| rs4402960 | 3 | IGF2BP2 | -0.03 | 0.05 | 0.56 | 19411 | --+-+- | -0.02 | 0.06 | 0.79 | 8867 | --+-++ | -0.04 | 0.08 | 0.64 | 10522 | ----+- |
| rs1801282 | 3 | PPARG | 0.05 | 0.07 | 0.43 | 19411 | +++-+- | -0.01 | 0.09 | 0.91 | 8867 | +++--+ | 0.12 | 0.11 | 0.28 | 10522 | +-+++- |
| rs11708067 | 3 | ADCY5 | 0.10 | 0.05 | 0.07 | 19411 | +++++- | 0.13 | 0.07 | 0.08 | 8867 | ++-++- | 0.04 | 0.08 | 0.66 | 10522 | +-+++- |
| rs4607103 | 3 | ADAMTS9 | 0.02 | 0.05 | 0.68 | 19411 | -+++-+ | 0.02 | 0.07 | 0.74 | 8867 | -++--+ | 0.01 | 0.08 | 0.87 | 10522 | --++-+ |
| rs1801214 | 4 | WFS1 | 0.09 | 0.05 | 0.05 | 19411 | ++++-+ | 0.10 | 0.06 | 0.11 | 8867 | ++-+-+ | 0.11 | 0.07 | 0.15 | 10522 | +-+--+ |
| rs4457053 | 5 | ZBED3 | 0.03 | 0.05 | 0.56 | 19411 | ++--++ | 0.08 | 0.07 | 0.24 | 8867 | ++-+-+ | -0.04 | 0.08 | 0.61 | 10522 | +---++ |
| rs7754840 | 6 | CDKAL1 | -0.01 | 0.05 | 0.87 | 19411 | +-++-+ | 0.03 | 0.06 | 0.65 | 8867 | +-++-- | -0.04 | 0.08 | 0.56 | 10522 | +--+-+ |
| rs864745 | 7 | JAZF1 | -0.02 | 0.04 | 0.73 | 19411 | ++---+ | 0.07 | 0.06 | 0.25 | 8867 | +++--+ | -0.12 | 0.07 | 0.09 | 10522 | +----+ |
| rs4607517 | 7 | GCK | -0.07 | 0.06 | 0.23 | 19411 | ----+- | -0.08 | 0.08 | 0.31 | 8867 | -+---- | -0.07 | 0.09 | 0.44 | 10522 | ----+- |
| rs972283 | 7 | KLF14 | -0.06 | 0.05 | 0.23 | 19411 | +----- | -0.06 | 0.06 | 0.31 | 8867 | +----+ | -0.06 | 0.07 | 0.43 | 10522 | ++-+-- |
| rs2191349 | 7 | DGKB/TMEM195 | -0.01 | 0.04 | 0.88 | 19411 | --+--+ | 0.00 | 0.06 | 0.99 | 8867 | --+-+- | -0.02 | 0.07 | 0.78 | 10522 | --++-+ |
| rs13266634 | 8 | SLC30A8 | -0.04 | 0.05 | 0.42 | 19411 | ----++ | -0.05 | 0.06 | 0.42 | 8867 | -----+ | -0.01 | 0.08 | 0.86 | 10522 | ----++ |
| rs896854 | 8 | TP53INP1 | -0.04 | 0.04 | 0.42 | 19411 | +--++- | 0.02 | 0.06 | 0.73 | 8867 | +-+++- | -0.11 | 0.07 | 0.12 | 10522 | ---++- |
| rs10811661 | 9 | CDKN2A/B | -0.05 | 0.06 | 0.43 | 19411 | -+-++- | 0.02 | 0.08 | 0.76 | 8867 | -+-++- | -0.14 | 0.09 | 0.12 | 10522 | -+---- |
| rs13292136 | 9 | TLE4 | -0.07 | 0.09 | 0.46 | 19411 | ------ | -0.02 | 0.11 | 0.88 | 8867 | -+---- | -0.11 | 0.14 | 0.42 | 10522 | ---++- |
| rs7903146 | 10 | TCF7L2 | 0.01 | 0.05 | 0.89 | 19411 | -++--- | -0.04 | 0.06 | 0.54 | 8867 | +++--- | 0.09 | 0.07 | 0.24 | 10522 | -+++-- |
| rs1111875 | 10 | HHEX | 0.00 | 0.05 | 0.99 | 19411 | -+-+-- | 0.03 | 0.06 | 0.60 | 8867 | ++-++- | -0.04 | 0.07 | 0.54 | 10522 | -+++-- |
| rs12779790 | 10 | CDC123/CAMK1D | 0.15 | 0.06 | 0.01 | 19411 | ++++++ | 0.12 | 0.08 | 0.13 | 8867 | +++++- | 0.17 | 0.10 | 0.07 | 10522 | ++++++ |
| rs2237892 | 11 | KCNQ1 | 0.08 | 0.09 | 0.37 | 19411 | --++++ | 0.07 | 0.12 | 0.55 | 8867 | --++++ | 0.13 | 0.15 | 0.38 | 10522 | --++++ |
| rs5219 | 11 | KCNJ11 | 0.18 | 0.11 | 0.12 | 2237 | ???+?? | 0.22 | 0.12 | 0.07 | 1656 | ???+?? | -0.06 | 0.27 | 0.83 | 581 | ???-?? |
| rs757110 | 11 | ABCC8 | -0.05 | 0.05 | 0.32 | 19411 | ++---- | -0.05 | 0.06 | 0.40 | 8867 | -+---+ | -0.06 | 0.07 | 0.43 | 10522 | +-++-- |
| rs1552224 | 11 | CENTD2 | -0.05 | 0.06 | 0.37 | 19411 | -++--- | -0.06 | 0.08 | 0.42 | 8867 | -+---- | -0.06 | 0.09 | 0.54 | 10522 | +++--- |
| rs10830963 | 11 | MTNR1B | -0.02 | 0.05 | 0.66 | 19411 | -+-+-- | -0.02 | 0.07 | 0.75 | 8867 | ++--+- | -0.03 | 0.08 | 0.68 | 10522 | --++-- |
| rs231362 | 11 | KCNQ1 | -0.01 | 0.05 | 0.91 | 19411 | -+-+-- | -0.06 | 0.06 | 0.33 | 8867 | -+-+-- | 0.07 | 0.08 | 0.33 | 10522 | -+++++ |
| rs1531343 | 12 | HMGA2 | -0.11 | 0.08 | 0.18 | 19411 | --+--- | -0.10 | 0.10 | 0.31 | 8867 | ---+-- | -0.07 | 0.12 | 0.56 | 10522 | --+-+- |
| rs7961581 | 12 | TSPAN8/LGR5 | 0.01 | 0.05 | 0.86 | 19411 | -++-+- | 0.04 | 0.07 | 0.57 | 8867 | -++-++ | -0.04 | 0.08 | 0.65 | 10522 | -++++- |
| rs7957197 | 12 | OASL/HNF1A | 0.01 | 0.05 | 0.85 | 19411 | -+++-- | 0.07 | 0.07 | 0.32 | 8867 | -+++-+ | -0.08 | 0.09 | 0.39 | 10522 | --++-- |
| rs8042680 | 15 | PRC1 | -0.06 | 0.05 | 0.19 | 19411 | ----+- | -0.08 | 0.06 | 0.21 | 8867 | ----+- | -0.04 | 0.08 | 0.59 | 10522 | --+-+- |
| rs11634397 | 15 | ZFAND6 | -0.03 | 0.05 | 0.54 | 19411 | ++-+-- | 0.06 | 0.06 | 0.36 | 8867 | -+-+++ | -0.17 | 0.08 | 0.03 | 10522 | +--+-- |
| rs8050136 | 16 | FTO | -0.09 | 0.05 | 0.06 | 19411 | ------ | -0.09 | 0.06 | 0.15 | 8867 | +---+- | -0.09 | 0.07 | 0.21 | 10522 | ---+-- |
| rs757210 | 17 | HNF1B | 0.00 | 0.05 | 0.97 | 19411 | -----+ | 0.01 | 0.07 | 0.83 | 8867 | ----++ | -0.04 | 0.08 | 0.62 | 10522 | ++---+ |
| *Direction order: CHS, AGES, ARIC, BMES, MESA, RS | | | | | | |  |  |  |  |  |  |  |  |  |  |  |
