# Supplementary material for: Genome-Wide Association Study of Retinopathy in Individuals without Diabetes
Source: PLoS One. 2013 Feb 5;8(2):e54232. doi: 10.1371/journal.pone.0054232 (PMC3564946; doi:10.1371/journal.pone.0054232)
Supplement: Table S5 — Meta-analysis results in SNPs associated with SBP, DBP and HTN. (DOCX) [file pone.0054232.s012.docx]

| Table S5, meta-analysis results in SNPs associated with SBP, DBP and HTN. | | | | | | | | | | | | | |  |  |  |  |  |
| --- | --- | --- | --- | --- | --- | --- | --- | --- | --- | --- | --- | --- | --- | --- | --- | --- | --- | --- |
|  |  |  |  | All Subjects | | | | | Subjects with Hypertension | | | | | Subjects without Hypertension | | | | |
| SNP | Chr | Locus |  | beta | se | p | n | direction | beta | se | p | n | direction | beta | se | p | n | Direction* |
| rs17367504 | 1 | MTHFR/  CLCN6/  NPPA/  NPPB/ | SBP | -0.07 | 0.06 | 0.26 | 19411 | +--++- | -0.14 | 0.08 | 0.07 | 8867 | +---+- | 0.02 | 0.10 | 0.87 | 10522 | +++++- |
| rs9815354 | 3 | ULK4 | DBP | -0.04 | 0.06 | 0.49 | 19411 | --+--- | -0.03 | 0.08 | 0.73 | 8867 | -+++-- | -0.05 | 0.10 | 0.60 | 10522 | +-+-+- |
| rs16998073 | 4 | PRDM8/  FGF5/  c4orf22 |  | 0.07 | 0.06 | 0.26 | 19411 | ++++-- | 0.11 | 0.08 | 0.14 | 8867 | +++++- | -0.02 | 0.10 | 0.85 | 10522 | -++--- |
| rs1530440 | 10 | c10orf107 | DBP | 0.00 | 0.06 | 0.96 | 19411 | -+-+++ | 0.03 | 0.07 | 0.67 | 8867 | -+-+-- | -0.03 | 0.09 | 0.72 | 10522 | +---++ |
| rs1004467 | 10 | TMEM26/RTKN2/  RHOBTB1/ARID5B | SBP | -0.09 | 0.08 | 0.22 | 19411 | --+++- | -0.07 | 0.10 | 0.48 | 8867 | --+++- | -0.13 | 0.12 | 0.28 | 10522 | --++-- |
| rs11191548 | 10 | CYP17A1 | SBP | -0.09 | 0.08 | 0.29 | 19411 | ---++- | -0.01 | 0.11 | 0.90 | 8867 | --+++- | -0.19 | 0.12 | 0.12 | 10522 | ---++- |
| rs1004467 | 10 | AS3MT/  CNNM2/  NT5C2 | SBP | -0.09 | 0.08 | 0.22 | 19411 | --+++- | -0.07 | 0.10 | 0.48 | 8867 | --+++- | -0.13 | 0.12 | 0.28 | 10522 | --++-- |
| rs11014166 | 10 | CACNB2 | DBP | 0.09 | 0.05 | 0.05 | 19411 | ++++-+ | 0.00 | 0.06 | 0.97 | 8867 | +++--+ | 0.22 | 0.08 | 4.3E-03 | 10522 | --++++ |
| rs381815 | 11 | PLEKHA7 | SBP | 0.02 | 0.05 | 0.66 | 19411 | -++-+- | 0.01 | 0.07 | 0.83 | 8867 | -+--+- | 0.04 | 0.08 | 0.60 | 10522 | -+++++ |
| rs2681492 | 12 | ATP2B1 | SBP | 0.11 | 0.06 | 0.08 | 19411 | ++++++ | 0.04 | 0.08 | 0.58 | 8867 | ++++-- | 0.21 | 0.10 | 0.03 | 10522 | ++++++ |
| rs2681472 | 12 | ATP2B1 | DBP/  HTN | 0.08 | 0.06 | 0.21 | 19411 | ++++-- | 0.02 | 0.08 | 0.80 | 8867 | ++++-- | 0.17 | 0.10 | 0.09 | 10522 | ++++++ |
| rs653178 | 12 | SHS2B3 | DBP | -0.04 | 0.04 | 0.40 | 19411 | ----++ | -0.09 | 0.06 | 0.12 | 8867 | ----++ | 0.03 | 0.07 | 0.64 | 10522 | ----++ |
| rs3184504 | 12 | ATXN2 | SBP/  DBP | 0.04 | 0.05 | 0.32 | 19411 | ++++-- | 0.10 | 0.06 | 0.10 | 8867 | ++++-- | -0.02 | 0.07 | 0.74 | 10522 | ++++-- |
| rs2384550 | 12 | TBX3/  TBX5 | DBP | -0.01 | 0.05 | 0.85 | 19411 | --++-+ | 0.05 | 0.06 | 0.45 | 8867 | --++++ | -0.10 | 0.07 | 0.19 | 10522 | --+--- |
| rs1378942 | 15 | CYP1A1 | DBP | -0.13 | 0.05 | 4.1E-03 | 19411 | ------ | -0.15 | 0.06 | 0.01 | 8867 | ------ | -0.12 | 0.07 | 0.12 | 10522 | ++---- |
| rs6495122 | 15 | CYP1A2/CSK/  LMAN1L/CPLX3/  ARID3B/  ULK3 | DBP | 0.08 | 0.05 | 0.07 | 19411 | +-++++ | 0.10 | 0.06 | 0.08 | 8867 | +++++- | 0.08 | 0.07 | 0.28 | 10522 | ---+++ |
| rs16948048 | 17 | ZNF652/PHB | DBP | 0.01 | 0.05 | 0.77 | 19411 | --+++- | -0.05 | 0.06 | 0.37 | 8867 | ---++- | 0.11 | 0.07 | 0.13 | 10522 | -+++++ |
| rs12946454 | 17 | PLCD3/  ABCD4/  HEXIM1/  HEXIM2 | SBP | -0.10 | 0.05 | 0.05 | 19411 | ------ | -0.10 | 0.06 | 0.13 | 8867 | --+--- | -0.10 | 0.08 | 0.21 | 10522 | ---+-- |
| *Direction order: CHS, AGES, ARIC, BMES, MESA, RS; SBP: Systolic Blood Pressure, DBP: Diastolic Blood Pressure, HTN: Hypertension | | | | | | | | | | | | | | | | | | |
