# Supplementary material for: Genome-Wide Association Study of Retinopathy in Individuals without Diabetes
Source: PLoS One. 2013 Feb 5;8(2):e54232. doi: 10.1371/journal.pone.0054232 (PMC3564946; doi:10.1371/journal.pone.0054232)
Supplement: Table S6 — Meta-analysis results in SNPs associated with diabetic retinopathy. (DOCX) [file pone.0054232.s013.docx]

| Table S6, meta-analysis results in SNPs associated with diabetic retinopathy. | | | | | | | | | | | | |  |  |  |  |  |
| --- | --- | --- | --- | --- | --- | --- | --- | --- | --- | --- | --- | --- | --- | --- | --- | --- | --- |
|  |  |  | All Subjects | | | | | Subjects with Hypertension | | | | | Subjects without Hypertension | | | | |
| SNP | Chr | Locus | beta | se | p | n | direction | beta | se | p | n | direction | beta | se | p | n | Direction* |
| rs6128 | 1 | *SELP* | -0.03 | 0.06 | 0.69 | 19411 | ---+++ | 0.01 | 0.08 | 0.92 | 8867 | ----++ | -0.06 | 0.10 | 0.52 | 10522 | ---+++ |
| rs6856425 | 4 | *IDUA* | -0.08 | 0.16 | 0.60 | 19411 | +-+-+- | -0.19 | 0.20 | 0.32 | 8373 | ?----- | 0.06 | 0.26 | 0.80 | 10522 | +-+-++ |
| *Direction order: CHS, AGES, ARIC, BMES, MESA, RS | | | | | | |  |  |  |  |  |  |  |  |  |  |  |
